# The amyloid-beta wave hypothesis of Alzheimer’s disease

**DOI:** 10.3389/fcimb.2025.1723095

**Published:** 2025-11-25

**Authors:** Sean J. Miller, Robert Logan, Can Zhang, Brian P. Hafler

**Affiliations:** 1 Department of Ophthalmology and Visual Science, Yale School of Medicine, New Haven, CT, United States; 2 The Alzheimer’s Pathobiome Initiative, Wake Forest, NC, United States; 3 Department of Biology and Biotechnology, School of Science and Technology, Endicott College, Beverly, MA, United States; 4 Genetics and Aging Research Unit, McCance Center for Brain Health, MassGeneral Institute for Neurodegenerative Disease, Department of Neurology, Massachusetts General Hospital and Harvard Medical School, Charlestown, MA, United States; 5 Department of Pathology, Yale School of Medicine, New Haven, CT, United States; 6 The Broad Institute, Massachusetts Institute of Technology and Harvard, Cambridge, MA, United States

**Keywords:** amyloid-beta, microbial infections, Alzheimer’s disease, aging, etiology, antimicrobial peptide

## Abstract

Alzheimer’s disease (AD) is a complex and multifactorial disorder that affects all races and genders. Genetic traits influenced by lifestyle and environment lead to a tremendous amount of heterogeneity in Alzheimer’s disease onset and severity. Regardless of these unique contributing factors, Alzheimer’s disease is traditionally met with amyloid-beta plaque formation in the central nervous system. In this commentary, we shed light on the growing literature surrounding amyloid-beta’s ability to act as an antimicrobial peptide in the central nervous system’s innate immune response to pathogenic infections. We hypothesize that there are, “amyloid-beta waves” that are created by the responses of neuroglia and neurons to microbial pathogens. The improper clearance and residual buildup of amyloid-beta waves throughout life increases the likelihood of developing Alzheimer’s disease. In conclusion, we suggest that anti-amyloid therapies during pathogenic infections or flare-ups may slow the development of Alzheimer’s disease by reducing amyloid-beta waves throughout the aging of individuals.

## Introduction

Alzheimer’s disease (AD) is a neurodegenerative disorder that affects millions of individuals worldwide. This progressive disease is characterized by cognitive and functional impairment, leading to a decline in episodic and working memory, executive function, and participation in activities of daily living. The underlying pathological complexity of AD arises from the interaction of various genetic and environmental factors.

Genetic predisposition for the development of AD, especially when linked to family history, yields a higher likelihood of developing AD. For example, the presence of certain genetic mutations, such as mutations in the presenilin 1 (PSEN1) or presenilin 2 (PSEN2) genes has been linked to early onset of AD. Furthermore, the apolipoprotein E4 (APOE4) gene variant has been identified as a major genetic risk factor, with almost all homozygotes exhibiting pathology, for late onset AD (Willett et al., 2025). These genetic predispositions contribute to the formation of amyloid-beta plaques, which are a hallmark histological feature of AD. Amyloid-beta plaques are formed when amyloid precursor protein is cleaved by the enzymes, beta-secretase and gamma-secretase, to produce amyloid-beta species. The fragments that result from this cleavage, including amyloid-beta peptide, are pathological species that aggregate to form plaques. The accumulation of amyloid-beta plaques triggers a cascade of inter- and intra-neuronal events that lead to cellular dysfunction, neurodegeneration, and cognitive decline.

The amyloid-beta pathology has led to the amyloid hypothesis of Alzheimer’s disease, originally published in 1992 (Hardy and Higgins, 1992). The amyloid hypothesis suggests amyloid-beta plaques are the initiator of Alzheimer’s disease pathogenesis. The downstream consequences illustrate that amyloid-beta dyshomeostasis induces cognitive impairment, tauopathy, gliosis, and blood-brain-barrier breakdown (Hampel et al., 2021). However, the amyloid hypothesis remains controversial, as anti-amyloid therapies have not improved cognition and the identification of postmortem human donors with amyloidopathy but no cognitive impairment (Kurkinen et al., 2023). These controversial reports warrant attention to the variability in disease progression severity (e.g., Tau and amyloid-beta pathology) among the heterogeneous individuals studied, as well as to the findings suggesting that removal of amyloid-beta may indeed slow the progression of Alzheimer’s disease (Sevigny et al., 2016; Jack et al., 2018; van Dyck et al., 2023). Collectively, these findings encourage further comprehension of the clinical heterogeneity of amyloid-beta in Alzheimer’s disease patients and how it pertains to the central nervous system’s microbial defensive mechanisms in those populations.

**Figure 1:**
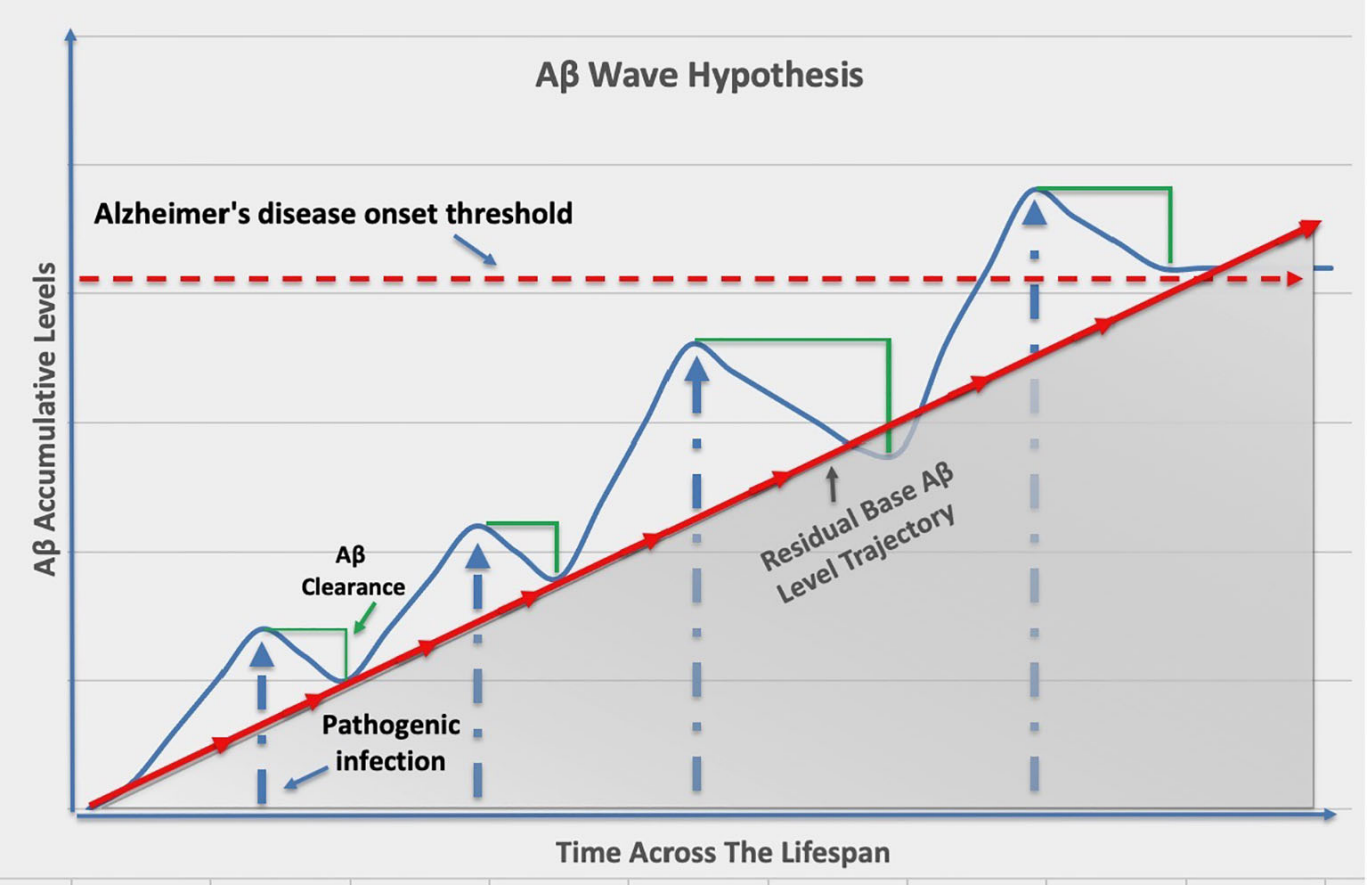
Repeated pathogenic infections throughout aging leads to increased amyloid-beta residual levels that raises the risk of developing Alzheimer’s disease.

Interestingly, recent research has shed light on an additional and intriguing aspect of amyloid-beta peptides; beyond their role in plaque formation, amyloid-beta peptides have been suggested to act as antimicrobial peptides (Moir et al., 2018). Antimicrobial peptides are components of the innate immune system that help protect the host from microbial infections. The hypothesis that amyloid-beta peptides may serve as antimicrobial peptides in the central nervous system is gaining traction. It proposes that in response to microbial pathogens or infections, neuroglia and neurons may release amyloid-beta peptides as part of the innate immune response. The newly generated amyloid-beta peptides may then act to neutralize or limit the spread of pathogens such as coronavirus, herpes simplex virus, *Chlamydia pneumoniae*, *Borrelia*, *Porphyromonas gingivalis*, *Varicella zoster* virus, and *Candida albicans* within the central nervous system, prompting the concept of “amyloid-beta waves” (**Figure 1**) (Dominy et al., 2019; MacDonald, 1986; Hammond et al., 2010; Pisa et al., 2015; Itzhaki et al., 2016; Eimer et al., 2018; Wu et al., 2019; Bathini et al., 2024; Miller et al., 2025). The hypothesis suggests that during episodes of pathogenic infections or inflammatory responses, there is an increased production and release of amyloid-beta peptides by brain cells. These “amyloid-beta waves” may serve as a defensive mechanism to combat microbial invaders identified in Alzheimer’s disease. However, the improper clearance or resolution of these amyloid-beta waves over time, especially in the context of aging, may result in their accumulation in the central nervous system.

There is growing support for the increase in amyloid biomarkers during microbial infections. In 2025, Duff et al. found that SARS-CoV-2 infection was associated with a significant reduction in the ratio of amyloid-beta 42:40, which is similar to 4 years of aging (Duff et al., 2025). One additional microbial Alzheimer’s disease study focusing on the connection with the herpes simplex virus, illustrated the increase in cerebral amyloid-beta burden in the human aging brain using positron emission tomography (Cantero et al., 2024).

There are a growing number of translational murine models that are utilized to investigate microbial infections with amyloid-beta deposition. In the 5XFAD mouse model of Alzheimer’s disease, Kumar and colleagues injected *Salmonella typhimurium* into the brains of aged mice to illustrate the significant increase in amyloid-beta plaques which colocalized with the bacteria (Kumar et al., 2016). Also, in the 5XFAD mouse model, Wang et al. infected mice with the herpes simplex virus and found accelerated amyloid-beta deposition, gliosis, and cognitive defects (Wang et al., 2024). Lastly, treatment with *Candida albicans* in the 5XFAD mouse model showed the elevation in amyloid-beta levels that accumulated around the yeast cells (Wu et al., 2019). The authors then went on to demonstrate that clearance of the infection improved cognitive dysfunction (Wu et al., 2019). Collectively, *in vivo* Alzheimer’s disease tools such as the 5XFAD mouse model can serve as critical models to comprehend the influence of microbes on amyloid-beta deposition in the central nervous system.

It is known that amyloid-beta clearance mechanisms can be non-enzymatic and enzymatic involving cell-type and neuro-anatomical specific features that become more prone to error with age and microbial infection (Wasén et al., 2024). Throughout aging the persistent lack of clearance of amyloid-beta peptides could potentially contribute to the development and progression of Alzheimer’s disease. This is suggested by the increased risk for the development of dementia from patients with co-infections such as varicella zoster virus or herpes simplex virus (Shin et al., 2024). In high-risk Alzheimer’s disease populations, such as those with Down Syndrome, the impact of microbes in neuropathological progression is largely unknown. One could speculate that these individuals would elicit an exaggerated response or lack proper clearance mechanisms to handle the amyloid-beta waves but more evidence is needed and attempts are underway to understand this (Ahmad et al., 2016).

The usage of anti-viral medications in dementia is not a new topic. Ongoing clinical trials and studies are exploring the correlations between vaccines and Alzheimer’s disease. Recently, the shingles vaccine was shown to correlate with a decreased chance of dementia (Eyting et al., 2025). Other supportive studies illustrate the presence of periodontal microbes in the brains of Alzheimer’s disease donors and the increased risk of developing Alzheimer’s disease in humans with periodontal disease (Beydoun et al., 2020). Collectively, these findings support amyloid-beta aggregation as being a mechanism to protect against microbial pathogens in the central nervous system.

Provided the growing evidence around the interplay between microbes, amyloid-beta, and Alzheimer’s disease, it is imperative that studies and therapies be developed to address this. Almost everyone, if not all, have experienced pathogenic infections, but the consequences inside aging brains of the heterogenous population are largely a mystery. The ability for amyloid-beta deposits to form in progressive waves suggests the need to develop suitable methods detecting these waves and to define integral moments for clearance-enhancing therapies. Prospective clinical studies could include imaging modalities such as positron emission tomography or non-invasive retinal micrographs that detect and quantify amyloid-beta levels in infectious disease individuals and age-matched controls (Ngolab et al., 2021). The usage of anti-amyloid therapies accompanied with clinical imaging, during and post-infection may render definitive answers to the amyloid-beta wave hypothesis.

In addition to the influence of amyloid-beta waves in Alzheimer’s disease, future investigations should explore additional neurodegenerative proteinopathies that have been associated with microbial infections. For example, around 50% of Parkinson’s disease patients and exhibit some form of amyloid-beta co-pathology in the central nervous system (Irwin et al., 2013). Another potential synergistic antimicrobial response or consequence to amyloid-beta is TDP-43 pathology that is found in approximately 25% of Alzheimer’s disease patients (Forman et al., 2007). The amyloid-beta co-pathology with the Parkinson’s disease-related alpha-synuclein and pathological TDP-43 inclusions in the central nervous system needs to be further explored in the context of microbial infections. One approach would involve protein-specific fluorescence clinical imaging using non-invasive and publicly accessible modalities such as previously shown by *in vivo* and *ex vivo* retinal fundus autofluorescence microscopy using amyloid-beta (42)-specific, CRANAD-28 (Emptage et al., 2016; Ran et al., 2020; Miller et al., 2025). These imaging modalities to detect and quantify amyloid-beta, alpha-synuclein, and TDP-43 could render invaluable insight into the physiological properties and downstream consequences of microbial infections in the aging central nervous system.

Collectively, this perspective on the antimicrobial peptide hypothesis joined with the newly formed, amyloid-beta wave hypothesis, forces us to consider microbial infections and flare-ups as potential lifetime influencers of neurodegeneration, particularly in aging brains, in our fight against Alzheimer’s disease.

## Data Availability

The original contributions presented in the study are included in the article/supplementary material. Further inquiries can be directed to the corresponding author.
